# Audit of pre-transfusion testing practices in hospital blood banks across Tanzania: A national survey

**DOI:** 10.4102/ajlm.v15i1.2986

**Published:** 2026-01-17

**Authors:** Oscar F. Mwashiuya, Magdalena A. Lyimo, Deus J. Mogela, Abdu J. Bhombo, Julius L. Mwimo, Edwin J. Shewiyo, Beatrice N. Tingo

**Affiliations:** 1 Department of Operations, National Blood Transfusion Service, Dar es Salaam, United Republic of Tanzania; 2 Department of Haematology and Blood Transfusion, Faculty of Medicine, Muhimbili University of Health and Allied Sciences, Dar es Salaam, United Republic of Tanzania; 3 Department of Monitoring and Evaluation, National Blood Transfusion Service, Dar es Salaam, United Republic of Tanzania; 4 Department of Administration, National Blood Transfusion Service, Dar es Salaam, United Republic of Tanzania; 5 Department of Epidemiology and Biostatistics, Faculty of Public Health, Kilimanjaro Christian Medical University College, Moshi, United Republic of Tanzania; 6 Department of Quality Assurance, National Blood Transfusion Service, Moshi, United Republic of Tanzania

**Keywords:** pre-transfusion testing, blood transfusion, hospital blood banks, Tanzania, haemovigilance

## Abstract

**Background:**

Blood transfusion is a lifesaving procedure performed across all healthcare levels in Tanzania. Despite significant investment in blood collection and screening, hospital transfusion practices have received less attention. With haemovigilance systems still in development, the understanding of pre-transfusion testing quality remains limited.

**Objective:**

This study aimed to evaluate hospital blood bank practices in conducting pre-transfusion procedures prior to issuing blood for transfusion.

**Methods:**

This descriptive cross-sectional study was conducted from January 2024 to March 2024 in 31 referral hospitals in Tanzania. Data on facility characteristics, testing methods, staffing, and equipment were collected through a validated questionnaire. Data were analysed using STATA version 18. Descriptive statistics were used to present key findings whereby continuous variables were presented as means, and categorical variables were presented as frequencies and percentages.

**Results:**

Among 31 participating facilities, ABO and Rhesus blood group systems typing and cross-matching were universally available (100%), while antibody screening was available in 13 out of 31 facilities (42.0%). Two out of 31 facilities (6.5%) used the less sensitive tile method for ABO typing. Critical reagents including Anti-D (immunoglobulin G), Anti-Human Globulin, and polythene tubes were available in only 21 out of 31 facilities (67.7%). While all facilities had standard operating procedures (SOPs) for basic tests, blood warming SOPs were available in 9 (29.0%) and fresh frozen plasma thawing SOPs were available in 14 (45.2%) out of 31 facilities.

**Conclusion:**

Significant gaps exist in pre-transfusion testing capabilities, SOPs, and essential reagents in Tanzanian referral hospital blood banks. Addressing these shortcomings is crucial for improving transfusion safety and strengthening haemovigilance.

**What this study adds:**

This study presents the first national audit of hospital blood bank practices in Tanzania, highlighting major gaps in antibody screening and cross-matching, resource shortages such as key reagents, and quality management deficiencies. It offers evidence-based, phased recommendations to strengthen pre-transfusion testing and improve safety in resource-limited settings.

## Introduction

Blood transfusion is a critical therapeutic intervention that saves lives across all levels of healthcare delivery. The World Health Organization estimates that approximately 118.5 million blood donations are collected globally each year, with the majority being transfused into patients with life-threatening conditions.^1^ In sub-Saharan Africa, where the burden of conditions requiring transfusion such as severe anaemia, obstetric haemorrhage, and trauma is high, ensuring safe blood transfusion practices is paramount.^2^ Tanzania has made substantial progress in establishing a national blood transfusion service since its inception in 2004. The country has achieved decentralised blood collection at the council level, and centralised screening for transfusion-transmissible infections at zonal laboratories.^3^ However, the critical final step in the transfusion chain and pre-transfusion compatibility testing conducted at hospital blood banks has received limited attention, despite its crucial role in preventing potentially fatal haemolytic transfusion reactions.^4^ Pre-transfusion testing encompasses a series of immunohaematological procedures designed to ensure compatibility between donor blood and recipient. These tests include ABO/Rhesus (Rh) blood group typing, antibody screening, and cross-matching, which together form the cornerstone of transfusion safety.^5^ The importance of these procedures is underscored by international data showing that incompatible transfusions resulting from errors in pre-transfusion testing remain a leading cause of transfusion-related morbidity and mortality.^6^

In Tanzania, the 2020 Service Availability and Readiness Assessment revealed concerning gaps in transfusion services. Only 8% of surveyed health facilities offered blood transfusion services, with a readiness score of just 63%. More alarmingly, while blood grouping was available in 97% of facilities providing transfusion services, compatibility testing was available in only 51%.^7^ These findings suggest significant vulnerabilities in the transfusion safety chain. Moreover, previous studies in Tanzania have highlighted specific deficiencies in pre-transfusion testing practices. A clinical audit at Muhimbili National Hospital, the country’s largest tertiary facility, reported the absence of routine indirect antiglobulin testing (IAT), raising concerns about the detection of clinically significant immunoglobulin G (IgG) antibodies.^8^ This finding is particularly concerning given that patients requiring multiple transfusions, such as those with sickle cell disease, and multiparous women are at increased risk of alloimmunisation and subsequent transfusion reactions.^9^

The quality of pre-transfusion testing is further influenced by the methods employed. Manual testing methods, which predominate in resource-limited settings, are associated with higher error rates compared to automated systems.^10^ On the other hand, lack of enough reagents is reported to be the main cause of pre-transfusion testing.^11^ In addition, lack of compliance with standard operating procedures (SOPs) and procedural gaps are also linked to transfusion errors, as reported by a study in Nigeria.^12^ This reality necessitates robust quality control mechanisms and standardised procedures in settings where manual testing is the norm. Despite these known challenges, comprehensive data on pre-transfusion testing practices across Tanzanian hospital blood banks remain scarce. This knowledge gap hampers efforts to improve transfusion safety and develop targeted interventions. Therefore, this study aimed to audit pre-transfusion testing practices in referral hospital blood banks across Tanzania, focusing on test availability, methods used, equipment and reagent availability, and adherence to SOPs.

## Methods

### Ethical considerations

Ethical approval was obtained from the National Institute for Medical Research with certificate number NIMR/HQ/R.8a/Vol.IX/4287. Administrative permission was granted by the Ministry of Health’s Chief Medical Officer (reference number: HB.209/313/01/138). Written informed consent was obtained from all participating facility medical officers-in-charge. The study adhered to the principles of the Declaration of Helsinki. All data were anonymised, and facility identities were coded to ensure confidentiality. Participants were informed of their right to withdraw at any time without consequences.

### Study design and setting

This multi-centre, hospital-based descriptive cross-sectional study was conducted from January 2024 to March 2024 in zonal and regional referral hospital blood banks across mainland Tanzania. Tanzania’s healthcare system is organised hierarchically, with referral hospitals serving as tertiary care centres receiving patients from lower-level facilities. These hospitals were selected as they handle the majority of complex cases requiring blood transfusion and are expected to maintain higher standards of laboratory services.

### Study population and sampling

A total of 34 referral hospitals (2 zonal referral hospitals and 32 regional referral hospitals) in mainland Tanzania were invited to participate. Inclusion criteria were: (1) operational hospital blood bank, (2) provision of blood transfusion services, and (3) willingness to participate. Exclusion criteria included facilities undergoing major renovation or those without functional blood banking services during the study period.

### Sample size determination

Given the finite population of referral hospitals and the audit nature of the study, we aimed for complete enumeration rather than statistical sampling. The target was to include all eligible referral hospitals to provide a comprehensive national picture of pre-transfusion testing practices.

### Data collection tool

A structured questionnaire (Online Supplementary Document 1) was developed based on the World Health Organization guidelines for blood transfusion services and Tanzania’s National Blood Transfusion Service standards. The 45-item questionnaire covered six domains: (1) facility characteristics (5 items, including hospital level, annual transfusion volume, catchment population), (2) pre-transfusion tests conducted (8 items, including types of tests, frequency of performance), (3) testing methods (6 items, including techniques used for each test type), (4) equipment and reagents (12 items, including availability and functionality), (5) quality management (10 items, including SOPs, documentation, quality control), and (6) staffing (4 items, including qualifications, training, task distribution). The questionnaire was pre-tested at two district hospitals (not included in final analysis) and refined based on feedback. Content validity was assessed by three blood banking experts, achieving a content validity index of 0.89.

### Data collection procedures

Data collection involved the following steps: ethical and administrative approvals were obtained from relevant authorities. Eight trained auditors from zonal blood transfusion centres underwent a two-day training on the study protocol and data collection tools. Hospital administrators were contacted two weeks prior to visit.

Auditors administered the questionnaire through face-to-face interviews with laboratory managers or designated blood bank personnel, combined with direct observation of practices and verification of equipment and reagent availability. Quality assurance was done whereby 10% of facilities were revisited by a different auditor to verify data accuracy and consistency.

### Data management and analysis

Data were entered into a password-protected MS Excel database with built-in validation checks. Double data entry was performed for 20% of questionnaires to assess entry accuracy. Data cleaning involved checking for completeness, consistency, and outliers. Statistical analysis was performed using Stata version 18 (StataCorp, College Station, Texas, United States). Descriptive statistics were calculated as follows: (1) continuous variables in mean and range, and (2) categorical variables in frequencies and proportions. Missing data were reported and excluded from percentage calculations.

### Data protection

All electronic data were encrypted and stored on password-protected computers. Physical documents were kept in locked cabinets accessible only to the research team. Data will be retained for five years post-publication before secure destruction.

## Results

### Response rate and facility characteristics

Of the 34 eligible referral hospitals, 31 participated in the audit, yielding a response rate of 91.2%. Three facilities were excluded: two because of ongoing renovation of laboratory services and one because of administrative challenges. The participating facilities comprised 29 regional referral hospitals (93.5%) and 2 zonal referral hospitals (6.5%). The participating hospitals collectively reported performing approximately 62 400 blood transfusions annually (mean: 1800 transfusions per facility; range: 480–8400). The facilities serve a combined catchment population of approximately 28 million people, representing about 60% of Tanzania’s mainland population.

### Availability of pre-transfusion tests

Table 1 presents the availability of pre-transfusion tests across participating facilities. Basic tests (ABO/Rh typing and cross-matching) were available in all facilities. With regard to advanced testing, Weak D testing was available in 74% of the facilities, Direct antiglobulin test was available in 61%, and antibody screening was available in 42% of the facilities.

**TABLE 1:** Availability of pre-transfusion tests in hospital blood banks, Tanzania mainland, January 2024 to March 2024 (*N* = 31).

Test	Currently conducted		Not conducted	
	*n*	%	*n*	%
ABO blood grouping	31	100	0	0
Rhesus typing	31	100	0	0
Cross-matching	31	100	0	0
Antibody screening	13	42.0	18	58.0
Direct antiglobulin test	19	61.3	12	38.7
Weak D testing	23	74.2	8	25.8
Antibody identification†	0	0	31	100

† , When positive antibody screens were encountered, samples were referred to the National Blood Transfusion Service for identification.

### Pre-transfusion test methods

The methods used for performing pre-transfusion tests varied across facilities. The majority of the facilities used manual tube testing for ABO/Rh typing (67.7%), and cross-matching (77%). Of the facilities providing antibody screening, the majority (76.9%) also used the manual tube testing method (Table 2). Three facilities reported using the less sensitive tile method for ABO/Rh typing and cross-matching. For about a quarter of facilities using mixed methods, the tile method was typically reserved for emergencies when rapid results were required. No facility reported having formal protocols defining when tile methods could be appropriately used.

**TABLE 2:** Methods used for performing pre-transfusion tests, Tanzania mainland, January 2024 to March 2024.

Test	Tube method		Tile method		Mixed methods		Total performing test	
	*n*	%	*n*	%	*n*	%	*n*	%
ABO/Rhesus typing	21	67.7	2	6.5	8	25.8	31	100
Antibody screening	10	76.9	0	0	3	23.1	13	100†
Cross-match	24	77.4	1	3.2	6	19.4	31	100

† , Denominator is 13 as only these facilities performed antibody screening.

### Cross-matching procedures

Further analysis of cross-matching procedures revealed variations in the completeness of testing: (1) immediate spin phase only, 10 facilities (32.3%); (2) immediate spin phase + 37 °C incubation, 7 facilities (22.6%); and (3) immediate spin + IAT phase, 14 facilities (45.1%).

This indicates that less than half of the facilities routinely performed the complete cross-match, including the IAT phase necessary for detecting clinically significant IgG antibodies.

### Availability of standard operating procedure and documentation

Table 3 summarises the availability of essential documents for blood banking practices. Standard operating procedures for ABO/Rh typing and cross-matching were available in all 31 facilities. The majority of facilities lacked SOPs for blood warming (71%), emergency blood release (61%), thawing fresh frozen plasma (54.8%), antibody screening (48.4%), and issuing blood (45.2%). For registers and forms, more than three-quarters of the facilities had all the essential forms available at the facility.

**TABLE 3:** Availability of guidelines, standard operating procedures, and registers, Tanzania mainland, January 2024 to March 2024 (*N* = 31).

Document	Available		Not available	
	*n*	%	*n*	%
**Standard operating procedures**				
SOP for ABO/Rhesus typing	31	100	0	0
SOP for cross-matching	31	100	0	0
SOP for antibody screening	16	51.6	15	48.4
SOP for weak D testing	23	74.2	8	25.8
SOP for issuing blood	17	54.8	14	45.2
SOP for blood warming	9	29.0	22	71.0
SOP for thawing FFP	14	45.2	17	54.8
SOP for emergency blood release	12	38.7	19	61.3
**Registers and forms**				
Blood bank compatibility register	29	93.6	2	6.4
Blood bank issuing register	29	93.6	2	6.4
Blood bank reception register	22	71.0	9	29.0
Blood transfusion consent form	28	90.3	3	9.7
Adverse event notification form	24	77.4	7	22.6
Transfusion reaction investigation form	24	77.4	7	22.6
Compatibility label	26	83.9	5	16.1

SOP, standard operating procedure; FFP, fresh frozen plasma.

### Equipment and reagent availability

Table 4 presents the availability of essential equipment needed for pre-transfusion testing. Regarding the availability of equipment, about 29% of the facilities lacked an alarm on the refrigerator, with 9.7% having a non-functional alarm; 12.9% of the facilities lacked a timer. Table 5 and Table 6 present the availability of essential reagents required for pre-transfusion testing. All facilities had ABO and Rh typing reagents, as presented in Table 5. On the other hand, the majority (64.5%) of the facilities lacked low ionic strength saline and enhancement media, and of the facilities providing antibody screening, only 42% had screening cells available (Table 6).

**TABLE 4:** Availability of essential equipment, Tanzania mainland, January 2024 to March 2024 (*N* = 31).

Equipment	Available		Non-functional		Not available	
	*n*	%	*n*	%	*n*	%
Bench-top centrifuge	28	90.3	2	6.5	1	3.2
Water bath	29	93.6	1	3.2	1	3.2
Refrigerator (2 °C – 6 °C)	31	100	0	0	0	0
Timer	27	87.1	0	0	4	12.9
Thermometer	30	96.8	0	0	1	3.2
Blood bank refrigerator with alarm	19	61.3	3	9.7	9	29.0

**TABLE 5:** Reagents which were universally available, Tanzania mainland, January 2024 to March 2024.

Reagent or supply	Available		Not available	
	*n*	%	*n*	%
Anti-A	31	100	0	0
Anti-B	31	100	0	0
Anti-AB	31	100	0	0
Anti-D (IgM or IgM + IgG)	31	100	0	0
Normal saline	31	100	0	0

Ig, immunoglobulin.

**TABLE 6:** Availability of other reagents and supplies, Tanzania mainland, January 2024 to March 2024 (*N* = 31).

Reagent or supply	Available		Intermittent supply		Not available	
	*n*	%	*n*	%	*n*	%
Anti-D (IgG only)	21	67.7	6	19.4	4	12.9
Anti-human globulin	21	67.7	5	16.1	5	16.1
Antibody screening cells	13	42.0	8	25.8	10	32.3
LISS or enhancement media	8	25.8	3	9.7	20	64.5
Polythene test tubes	21	67.7	7	22.6	3	9.7
Glass test tubes	31	100	0	0	0	0
Pasteur pipettes	31	100	0	0	0	0

Ig, immunoglobulin; LISS, low ionic strength saline.

### Staffing patterns

All facilities reported having at least one laboratory scientist (bachelor’s degree holder), with an average of three laboratory scientists per facility. The majority (96.8%) had a laboratory technologist (diploma), with an average of five per facility. Only 14 (45%) of the facilities had a dedicated blood bank staff, with an average of two staff members per facility (Table 7).

**TABLE 7:** Staffing categories in hospital blood banks, Tanzania mainland, January 2024 to March 2024 (*N* = 31).

Staff category	Facilities with staff		Mean per facility	
	*n*	%	Mean	Range
Laboratory Scientist (BSc)	31	100	3.2	1–8
Laboratory Technologist (Diploma)	30	96.8	5.4	0–12
Laboratory Assistant (Certificate)	19	61.3	2.1	0–6
Dedicated blood bank staff†	14	45.2	1.8	0–4

BSc, Bachelor of Science degree.

† , Staff exclusively assigned to blood bank duties.

However, the distribution of staff categories and their involvement in pre-transfusion testing varied. Task distribution analysis revealed that in 12 facilities (38.7%), laboratory assistants were involved in performing pre-transfusion tests despite national guidelines restricting this practice. This was more common in facilities with high transfusion volumes and limited senior staff.

### Quality control practices

Quality control practices varied significantly across facilities: (1) daily positive and negative controls for ABO/Rh, 18 facilities (58.1%); (2) documented temperature monitoring, 22 facilities (71.0%); (3) participation in external quality assessment, 11 facilities (35.5%); and (4) regular internal audits, 7 facilities (22.6%).

### Training and competency

Analysis of staff training revealed: (1) formal blood banking training in the past two years, 42.3% of staff; (2) on-the-job training only, 38.5% of staff; (3) no specific blood banking training, 19.2% of staff.

## Discussion

This comprehensive audit of pre-transfusion testing practices in Tanzanian referral hospitals reveals both achievements and significant gaps in blood transfusion safety. While basic ABO/Rh typing and cross-matching are universally available, the limited implementation of antibody screening and complete cross-matching procedures poses substantial risks to transfusion recipients.

### Critical gaps in antibody detection

The finding that only 42% of facilities routinely perform antibody screening is particularly concerning. This aligns with previous findings from Muhimbili National Hospital,^8^ and suggests a systemic issue rather than isolated deficiencies. Antibody screening is essential for detecting unexpected alloantibodies that can cause severe haemolytic transfusion reactions.^13^ The absence of this test is especially problematic in populations with high transfusion requirements, such as sickle cell disease patients who are prevalent in Tanzania.^14^

Furthermore, among facilities performing cross-matching, less than half (45.1%) include the IAT phase necessary for detecting IgG antibodies. This incomplete testing may miss clinically significant antibodies, particularly in previously transfused patients or multiparous women.^15^ Studies from other African countries have reported alloimmunisation rates of 13.7% in multiply transfused patients,^16^ highlighting the importance of comprehensive antibody detection.

### Methodological concerns

The continued use of tile methods in some facilities, though limited, represents a significant quality concern.

The tile method’s lower sensitivity compared to tube methods can lead to false-negative results, potentially resulting in incompatible transfusions.^17^ The World Health Organization and international standards explicitly recommend against tile methods for routine testing.^18^ The fact that some facilities resort to tile methods during emergencies without formal protocols further compounds the risk.

### Resource and infrastructure limitations

The unavailability of critical reagents such as Anti-D (IgG), Anti-Human Globulin, and antibody screening cells in approximately one-third of the studied facilities directly impacts testing capabilities. This finding corroborates the Service Availability and Readiness Assessment 2020 report, which highlighted resource constraints in Tanzanian health facilities.^7^ Without these reagents, facilities cannot perform complete pre-transfusion testing regardless of staff competence or equipment availability.

The intermittent supply reported by several facilities (Table 5) suggests supply chain challenges that require systematic intervention. Previous studies in sub-Saharan Africa have identified similar reagent availability issues as major barriers to quality blood banking services.^19^ The Ministry of Health should prioritise enforcement of these reagents through the Medical Stores Department, which is the government procurement entity for medical supplies.

### Quality management system deficiencies

The absence of SOPs for critical procedures such as blood warming (71% lacking) and emergency blood release (61.3% lacking) indicates significant gaps in quality management systems. These findings echo broader challenges in laboratory quality management in resource-limited settings.^20^ The low participation in external quality assessment programmes (35.5%) further limits opportunities for performance improvement and benchmarking. The quality management system deficiencies indicate the presence of country-wide systematic challenges that need to be addressed from a high level at the Ministry of Health in order to enforce development of hospital blood bank quality management system and its implementation for patient safety.

### Staffing and competency challenges

While all facilities have qualified laboratory scientists, the involvement of laboratory assistants in pre-transfusion testing at 38.7% of facilities violates national guidelines and potentially compromises test quality.

This practice likely reflects workload pressures and staffing shortages common in African healthcare systems.^21^ The limited formal training in blood banking (only 42.3% of staff trained in the past two years) suggests inadequate investment in continuous professional development. The presence of laboratory assistants performing blood bank procedures poses questions on the quality of test results produced and this does not align with the national guidelines.

### Implications for patient safety

These findings have direct implications for transfusion safety in Tanzania. The combination of incomplete antibody detection, methodological weaknesses, and resource constraints creates multiple vulnerabilities in the transfusion chain. International data suggest that pre-transfusion testing errors contribute to approximately 1 in 19 000 transfusions resulting in acute haemolytic reactions.^22^ In settings with limited haemovigilance systems, the true incidence of transfusion reactions may be significantly underreported.^23^

### Comparison with regional standards

Compared to other East African countries, Tanzania’s pre-transfusion testing capabilities show both similarities and differences. A study from Kenya reported antibody screening availability in 58% of referral hospitals,^24^ slightly higher than our findings. However, Uganda reported similar challenges, where facilities were reported as not performing routine IAT crossmatch and antibody screening.^15^ These regional variations suggest opportunities for cross-border learning and standardisation.

### Recommendations

Based on these findings, we first recommend immediate actions to develop and implement a national algorithm for pre-transfusion testing that accounts for resource limitations while maintaining safety, establish minimum standards for emergency blood release procedures, and strengthen supply chain management for critical blood banking reagents. Second, we recommend immediate efforts to conduct targeted training on complete cross-matching procedures and implement regular internal quality audits in all hospital blood banks. Third, in the long term, the scale-up of a national external quality assessment programme for pre-transfusion testing, which currently covers 25% of all transfusing facilities.

### Limitations

This study has several limitations that should be taken into account when interpreting the findings. First, the focus on referral hospitals limits the generalisability of the results to lower-level facilities, where resource constraints may be more pronounced. District and health centre laboratories that also provide transfusion services are likely to experience even greater challenges. Second, the study did not include clinical correlation; the audit did not link laboratory findings to clinical outcomes such as transfusion reaction rates, which restricts our ability to assess the clinical implications of the identified gaps. Third, the study may be subject to social desirability bias, as data were collected by auditors and respondents may have overstated compliance with SOPs. Fourth, although auditors were trained and used a standardised checklist, inter-rater reliability (e.g., kappa) was not formally assessed, which may introduce potential variability in scoring.

Lastly, the cross-sectional design does not allow for the assessment of trends over time or changes in laboratory practices.

### Conclusion

This comprehensive audit reveals that while basic pre-transfusion testing infrastructure exists in Tanzanian referral hospitals, significant gaps in advanced testing capabilities, quality management systems, and resource availability compromise transfusion safety. The limited implementation of antibody screening and incomplete cross-matching procedures pose particular risks to frequently transfused patients. Addressing these deficiencies requires development and execution of an action plan to address quality management system implementation in hospital blood banks (SOPs, records, staff competency, and testing algorithm), equipment requirements, supply chain strengthening, and staff capacity building. Future research should focus on assessing pre-transfusion testing practices at lower-level facilities, establishing the clinical impact of current testing gaps through prospective haemovigilance studies, and a cost analysis in assessing the health system in blood banking services to understand financial implications of implementing recommended improvements, which is crucial for policy decisions. This audit provides essential baseline data to guide evidence-based interventions aimed at strengthening transfusion safety in Tanzania and similar settings.

## References

[1] World Health Organization. Global status report on blood safety and availability 2021. Geneva: WHO; 2022.

[2] Tagny CT, Mbanya D, Tapko JB, Lefrère JJ. Blood safety in sub-Saharan Africa: A multi-factorial problem. Transfusion (Paris). 2008;48(6):1256–1261. 10.1111/j.1537-2995.2008.01697.x 18713111

[3] Matee MIN, Magesa PM, Lyamuya EF. Seroprevalence of human immunodeficiency virus, hepatitis B and C viruses and syphilis infections among blood donors at the Muhimbili National Hospital in Dar Es Salaam, Tanzania. BMC Public Health. 2006;6:21. 10.1186/1471-2458-6-21 16445860 PMC1373616

[4] Bloch EM, Vermeulen M, Murphy E. Blood transfusion safety in Africa: A literature review of infectious disease and organizational challenges. Transfus Med Rev. 2006;26:164–180. 10.1016/j.tmrv.2011.07.006 PMC3668661 21872426

[5] Shulman IA, Downes KA, Sazama K, Maffei LM. Pretransfusion compatibility testing for red blood cell administration. Curr Opin Hematol. 2001;8:397–404. 10.1097/00062752-200111000-00014 11604582

[6] Bolton-Maggs PHB, Cohen H. Serious hazards of transfusion (SHOT) haemovigilance and progress is improving transfusion safety. Br J Haematol. 2013;163(3):303–314. 10.1111/bjh.12547 24032719 PMC3935404

[7] Ministry of Health Community Development, Gender, Elderly and Children. Tanzania service availability and readiness assessment report. Dodoma: Ministry of Health, Community Development, Gender, Elderly and Children; 2020.

[8] Makubi AN, Meda C, Magesa A, et al. Audit of clinical-laboratory practices in haematology and blood transfusion at Muhimbili national hospital in Tanzania. Tanzan J Health Res. 2012;14(4):1–8. 10.4314/thrb.v14i4.4 26591723 PMC5612394

[9] Natukunda B, Schonewille H, Ndugwa C, Brand A. Red blood cell alloimmunization in sickle cell disease patients in Uganda. Transfusion (Paris). 2010;50(1):20–25. 10.1111/j.1537-2995.2009.02435.x 19821949

[10] Mistry H, Poles D, Watt A, Bolton-Maggs PHB. Human errors in manual techniques for ABO/D grouping are associated with potentially lethal outcomes. Transfus Med. 2019;29(4):262–267. 10.1111/tme.12616 31309638

[11] Bugge HF, Karlsen NCT, Oydna E, et al. A study of blood transfusion services at a district hospital in Malawi. Vox Sang. 2013;104(1):37–45. 10.1111/j.1423-0410.2012.01628.x 22765350

[12] Aneke J, Okocha C. Blood transfusion safety; current status and challenges in Nigeria. Asian J Transfus Sci. 2017;11(1):1–5. 10.4103/0973-6247.200781 28316432 PMC5345273

[13] Tormey CA, Fisk J, Stack G. Red blood cell alloantibody frequency, specificity, and properties in a population of male military veterans. Transfusion (Paris). 2008;48:2069–2076. 10.1111/j.1537-2995.2008.01815.x 18631165

[14] Makani J, Cox SE, Soka D, et al. Mortality in sickle cell anemia in Africa: A prospective cohort study in Tanzania. PLoS One. 2011;6(2):e14699. 10.1371/journal.pone.0014699 21358818 PMC3040170

[15] Natukunda B, Ndeezi G, See Er L, Bajunirwe F, Teramura G, Delaney M. The role of improved pre-transfusion testing in the prevention of delayed serologic transfusion reactions among blood recipients in Uganda: A randomized controlled trial (IPAT Study). ISBT Sci Ser. 2019;14:366–373. 10.1111/voxs.12493

[16] Allali S, Peyrard T, Amiranoff D, et al. Prevalence and risk factors for red blood cell alloimmunization in 175 children with sickle cell disease in a French university hospital reference centre. Br J Haematol. 2017;177:641–647. 10.1111/bjh.14609 28402005

[17] White J. Pre-transfusion testing. ISBT Sci Ser. 2009;4:37–44. 10.1111/j.1751-2824.2009.01211.x

[18] World Health Organization. Safe blood and blood products: Manual on the management, maintenance and use of blood cold chain equipment. Geneva: World Health Organization: 2005.

[19] Kajja I, Bimenya G, Smit Sibinga C. The interface between blood preparation and use in Uganda. Vox Sang. 2010;98(3p1):e257–e262. 10.1111/j.1423-0410.2009.01296.x 20059759

[20] Alemnji GA, Zeh C, Yao K, Fonjungo PN. Strengthening national health laboratories in sub-Saharan Africa: A decade of remarkable progress. Trop Med Int Health. 2014;19(4):450–458. 10.1111/tmi.12269 24506521 PMC4826025

[21] Scheffler RM, Campbell J, Cometto G, et al. Forecasting imbalances in the global health labor market and devising policy responses. Hum Resour Health. 2018;16:5. 10.1186/s12960-017-0264-6 29325556 PMC5765602

[22] Hendrickson JE, Tormey CA. Understanding red blood cell alloimmunization triggers. Am Soc Hematol. 2016;2016(1):446–451. 10.1182/asheducation-2016.1.446 PMC6142457 27913514

[23] Samukange WT, Kluempers V, Porwal M, et al. Implementation and performance of haemovigilance systems in 10 sub-Saharan African countries is sub-optimal. BMC Health Serv Res. 2021;21:1258. 10.1186/s12913-021-07235-0 34801022 PMC8605544

[24] Mwangi JW, Kimani D, Oduor M. Approaches to advancing blood safety through haemovigilance: A review. East Afr Med J. 2009;86(12):S93–S97. 10.4314/eamj.v86i12.62915 21591517

